# Supplementary material for: Structural basis for the self-recognition of sDSCAM in Chelicerata
Source: Nat Commun. 2023 May 2;14:2522. doi: 10.1038/s41467-023-38205-1 (PMC10154414; doi:10.1038/s41467-023-38205-1)
Supplement: Supplementary file 1 — Supplementary Information [file 41467_2023_38205_MOESM1_ESM.pdf]

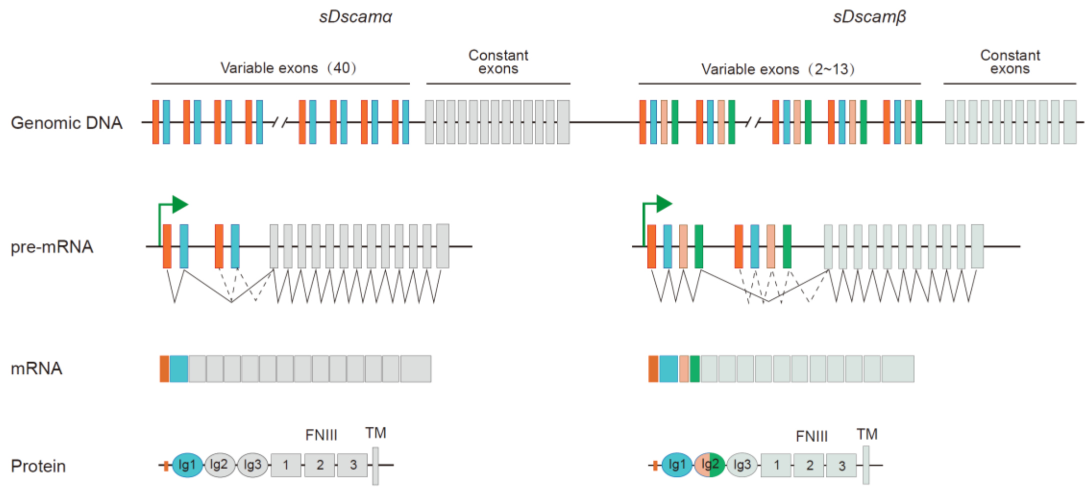

Supplementary Figure 1. Organization of the two *sDscam* gene subfamilies in *M. martensii*. One α and six β subfamilies are composed of multiple tandemly arrayed regions (colored boxes) and common regions (grey boxes). Each *sDscamα* variable cassette encodes an N-terminal fragment and Ig1 domain, while that of *sDscamβ* encodes an N-terminal fragment and Ig1–Ig2 domains.

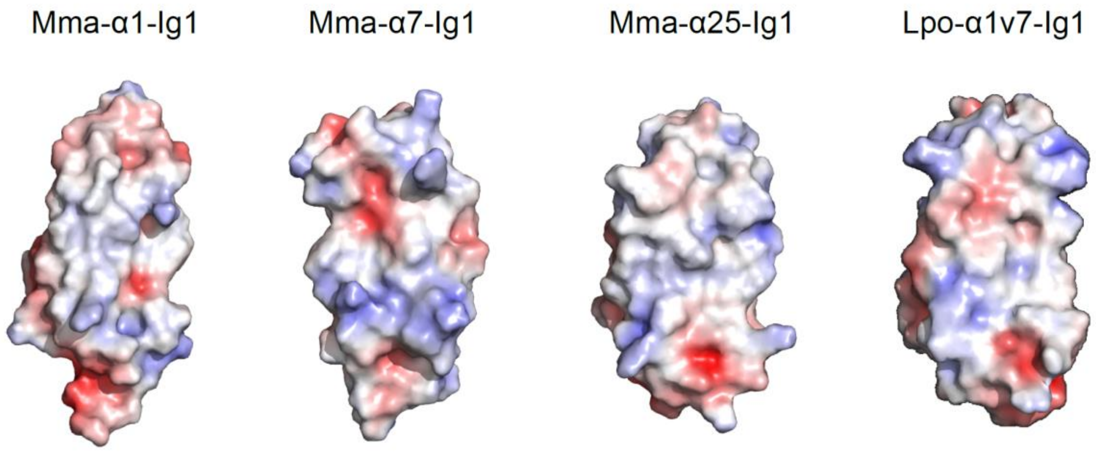

Supplementary Figure 2. The electrostatic surface potential of the ABED face of sDscamα Ig1 isoforms. Blue and red (±5 kT/e) indicate the positively and negatively charged areas of the protein, respectively.

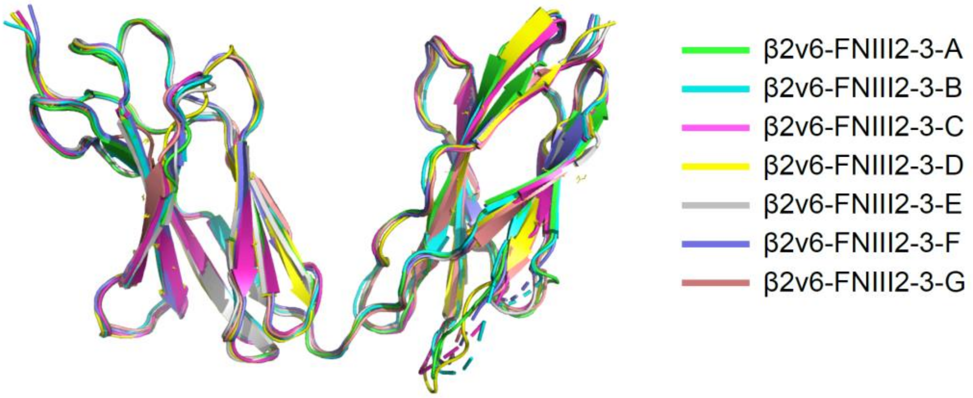

Supplementary Figure 3. Superposition of sDscam FNIII2-3 fragments. The seven molecules per asymmetric unit in the FNIII2-3 crystal structure of sDscam isoform β2v6 are superimposed, which reveals an almost identical conformation.

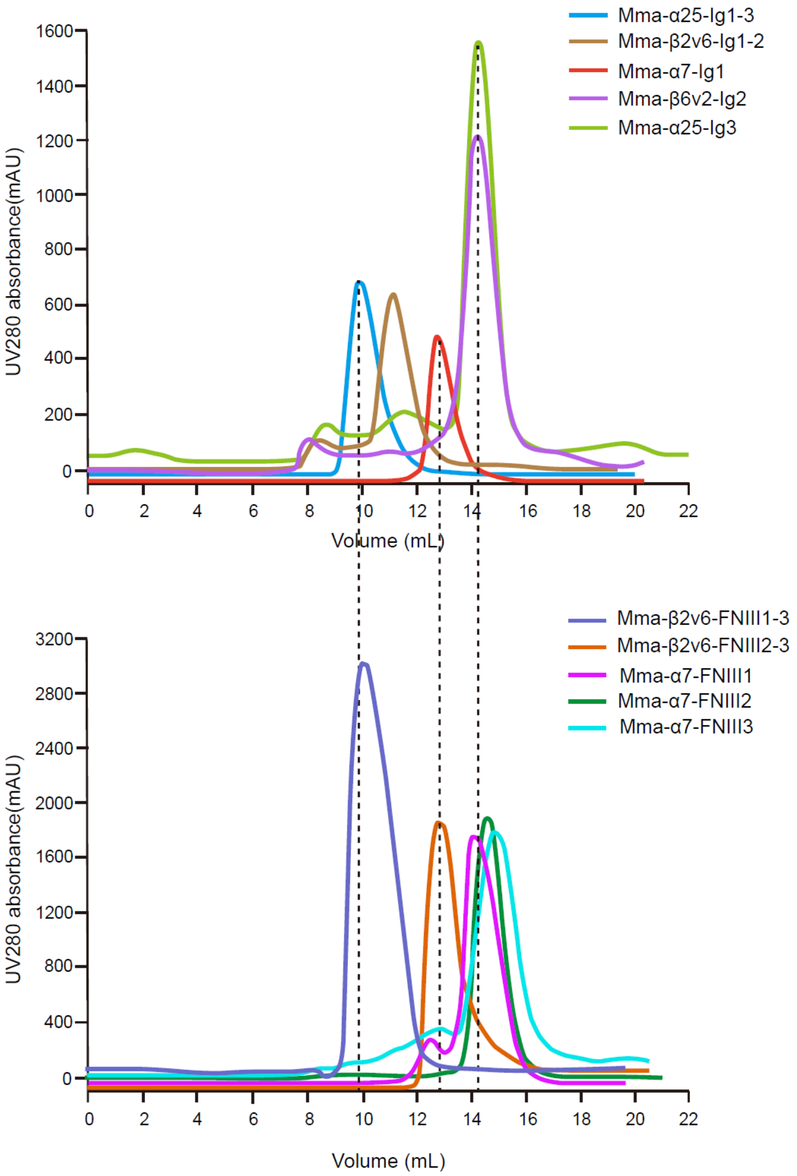

Supplementary Figure 4. Gel-filtration profiles of different sDscam fragments. Upper panel, the gel-filtration profiles of Ig fragments. Lower panel, the gel-filtration profiles of FNIII fragments. Dashed lines indicate the same elution volumes.

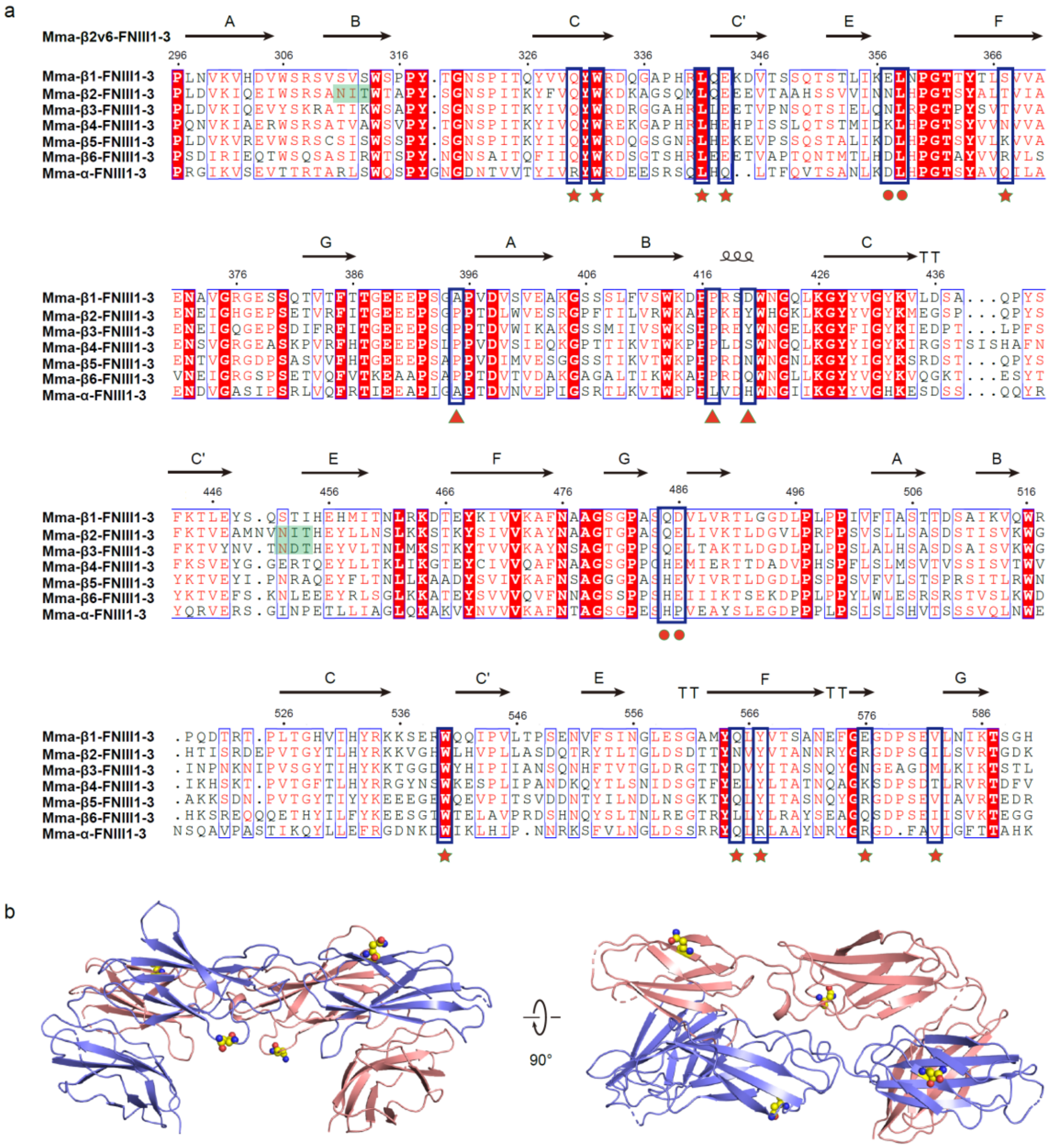

Supplementary Figure 5. Sequence alignment of *M. martensii* sDscam FNIII domains. (**a**) The sequence alignment of sDscam FNIII domains is generated with CLUSTAL_Omega (https://www.ebi.ac.uk/Tools/msa/clustalo/) and illustrated with ESPript (https://espript.ibcp.fr/ESPript/cgi-bin/ESPript.cgi/). The secondary structural elements and residue number are marked in accordance with sDscam isoform β2v6. Stars, filled circles and triangles below the sequences indicate the residues involved in the interactions of FNIII1-FNIII3, FNIII1-FNIII2 and FNIII2-FNIII2 interfaces, respectively. The potential N-linked glycosylation motifs (NXS/T) are shaded in green. (**b**) Visualization of the potential glycosylation sites on sDscam *cis*-dimer structure. The asparagine residues of the glycosylation sites are shown as spheres.

Supplementary Table 1. Crystallization conditions.

Proteins	Crystallization conditions
Mma-sDscam α1-Ig1	40% (v/v) 2-propanol, 100 mM imidazole/hydrochloric acid pH 6.5, 15% (w/v) PEG 8,000
Mma-sDscam α7-Ig1	0.2 M ammonium acetate, 0.1 M sodium citrate tribasic dihydrate pH 6.0, 25% (w/v) PEG 3,350, 5% (v/v) Jeffamine M-600® pH 7.0
Mma-sDscam β6v2-Ig1	0.2 M magnesium formate, 20% (w/v) PEG 3,350
Mma-sDscam β2v6-Ig1-2	0.2 M magnesium chloride, 0.1 M HEPES pH 7.0, 20% (w/v) PEG 6,000
Mma-sDscam α25-Ig1-3	0.1 M KSCN, 15% (w/v) PEG 2,000, 0.1 M glycine, 0.1 M Bis-Tris pH 6.0
Lpo-sDscam α1v7-Ig1	1 M Sodium citrate tribasic, 100 mM sodium cacodylate/ hydrochloric acid pH 6.5
Lpo-sDscam β3v7-Ig1	0.2 M $MgCl_2$, 0.1 M Tris pH 8.5, 20% (w/v) PEG 4,000
Mma-sDscam α7-FNIII1	25% (w/v) PEG 1,500, 100 mM SPG buffer pH 5.5
Mma-sDscam α7-FNIII2	0.1 M HEPES buffer pH 7.5, 1.5 M lithium sulfate monohydrate
Mma-sDscam α7-FNIII3	0.1 M NaH_2PO_4 pH 6.5, 18% (w/v) PEG 8,000, 3% (v/v) isopropanol
Mma-sDscam β2v6-FNIII23	0.2 M NaCl, 14% (w/v) PEG 8,000, 100 mM HEPES buffer pH 7.0
Mma-sDscam β2v6-FNIII123	20% (w/v) PEG 3,350, 100 mM PCB buffer pH 4.5

Supplementary Table 2. Data collection and refinement statistics.

**Construct**	α1-Ig1	α7-Ig1	β2v6-Ig1-2	β6v2-Ig1	α25-Ig1-3	α1v7-Ig1	β3v7-Ig1	α7-FNIII1	α7-FNIII2	α7-FNIII3	β2v6-FNIII2-3	β2v6-FNIII1-3
**PDB ID**	7Y54	7Y4X	7Y9A	7Y95	7Y6O	7Y5J	7Y73	7Y8H	7Y5R	7Y8I	7Y6E	7Y8S
**Data collection**												
Space group	$C2$	$P4_32_12$	$I\,2$	$P2_1$	$H32$	$P6_522$	$P2_1$	$C2$	$P2_12_12_1$	$P2_12_12_1$	$C2$	$P6_422$
Cell dimensions												
a, *b*, *c* (Å)	67.4, 40.7, 41.5	92.5, 92.5, 69.9	63.3, 49.0, 82.0	45.2, 39.1, 60.9	117.5, 117.5, 171.5	64.8, 64.8, 113.1	39.6, 40.9, 54.2	81.9, 43.2, 48.3	28.9, 53.3, 60.8	69.1, 85.8, 106.2	307.0, 56.2, 94.8	171.6, 171.6, 114.9
α, *β*, *γ* (°)	90.0, 94.2, 90.0,	90.0, 90.0, 90.0	90.0, 104.7, 90.0	90.0, 108.9 90.0	90.0, 90.0, 120.0	90.0, 90.0, 120.0	90.0, 100.1, 90.0	90.0, 111.3, 90.0	90.0, 90.0, 90.0	90.0, 90.0, 90.0	90.0, 105.4, 90.0	90.0, 90.0, 120.0
Resolution (Å)	50.00-1.80 (1.83-1.80)*	50.00-2.95 (3.00-2.95)	50.00-2.50 (2.54-2.50)	50.00-1.55 (1.58-1.55)	50.00-3.10 (3.17-3.10)	50.00-1.60 (1.63-1.60)	50.00-1.32 (1.36-1.32)	50.0-1.80 (1.84-1.80)	50.00-1.56 (1.60-1.56)	50.00-1.90 (1.93-1.90)	50.00-3.05 (3.10-3.05)	50.00-2.70 (2.75-2.70)
R_{pim} (%)	8.3 (11.9)	4.3 (34.5)	3.8 (9.9)	3.8 (32.7)	3.0 (26.1)	1.5 (17.1)	4.9 (11.8)	5.5 (28.4)	5.1 (13.4)	3.4 (38.1)	7.2 (43.3)	3.7 (38.2)
$I/\sigma I$	8.6 (6.5)	20.5 (2.0)	30.1 (6.9)	22.5 (1.7)	23.8 (2.0)	46.7 (4.0)	17.9 (1.8)	13.1 (2.2)	13.9 (10.0)	22.9 (2.0)	10.2 (1.7)	26.0 (2.0)
CC (1/2)	0.96 (0.94)	0.96 (0.74)	0.97 (0.97)	0.99 (0.82)	0.98 (0.90)	0.99 (0.94)	0.98 (0.87)	0.97 (0.78)	0.97 (0.95)	0.99 (0.67)	0.99 (0.72)	0.98 (0.79)
Completeness (%)	100.0 (99.8)	99.9 (99.4)	98.0 (96.6)	99.6 (97.9)	100.0 (99.6)	100.0(100.0)	98.1 (87.7)	99.3 (96.7)	100.0 (100.0)	99.8 (98.7)	98.7 (90.9)	100.0(100.0)
Redundancy	6.4 (6.1)	24.3 (17.4)	6.0 (5.1)	5.7 (4.1)	16.0 (12.2)	37.8 (35.9)	4.1 (2.7)	6.2 (4.2)	11.9 (9.7)	12.9 (10.7)	4.7 (3.8)	38.3 (31.8)
**Refinement**												
Unique reflections	10,339	6,660	8,285	30,014	7,501	19,192	35,491	14,018	13,650	46,459	25,974	25,039
$R_{work/}$ R_{free}	18.0/22.6	21.0/24.6	23.6/28.8	15.0/19.1	25.1/28.7	18.9/21.4	18.8/22.1	22.3/27.2	13.9/15.9	19.8/22.9	23.4/27.2	22.2/27.4
Ramachandran												
Favored (%)	96.94	98.94	96.28	98.45	94.35	98.92	99.47	95.43	99.03	98.23	97.75	97.27
Outlier (%)	0.00	0.00	0.00	0.00	0.71	0.00	0.00	0.57	0.00	0.00	0.00	0.00
R.m.s deviations												
Bond lengths (Å)	0.007	0.003	0.005	0.006	0.004	0.006	0.005	0.007	0.005	0.007	0.002	0.004
Bond angles (º)	0.745	0.757	1.008	0.718	0.763	0.906	0.786	0.759	0.738	0.862	0.644	0.688

*Highest resolution shell is shown in parenthesis.
