# Supplementary material for: Structural basis for the self-recognition of sDSCAM in Chelicerata
Source: Nat Commun. 2023 May 2;14:2522. doi: 10.1038/s41467-023-38205-1 (PMC10154414; doi:10.1038/s41467-023-38205-1)
Supplement: Supplementary file 3 — Description of additional supplementary files [file 41467_2023_38205_MOESM3_ESM.pdf]

Description of additional supplementary files

Supplementary Data 1 : Supplementary Data List of all PCR primers used in the study.
