# Supplementary material for: Structural basis for the self-recognition of sDSCAM in Chelicerata
Source: Nat Commun. 2023 May 2;14:2522. doi: 10.1038/s41467-023-38205-1 (PMC10154414; doi:10.1038/s41467-023-38205-1)
Supplement: Supplementary file 5 — Reporting Summary [file 41467_2023_38205_MOESM5_ESM.pdf]

nature portfolio

Corresponding author(s): Qiang Chen

Last updated by author(s): Apr 13, 2023

Reporting Summary

Nature Portfolio wishes to improve the reproducibility of the work that we publish. This form provides structure for consistency and transparency in reporting. For further information on Nature Portfolio policies, see our Editorial Policies and the Editorial Policy Checklist.

Statistics

For all statistical analyses, confirm that the following items are present in the figure legend, table legend, main text, or Methods section.

n/a	Confirmed	
☐	☒	The exact sample size (*n*) for each experimental group/condition, given as a discrete number and unit of measurement
☐	☒	A statement on whether measurements were taken from distinct samples or whether the same sample was measured repeatedly
☐	☒	The statistical test(s) used AND whether they are one- or two-sided<br>*Only common tests should be described solely by name; describe more complex techniques in the Methods section.*
☒	☐	A description of all covariates tested
☒	☐	A description of any assumptions or corrections, such as tests of normality and adjustment for multiple comparisons
☐	☒	A full description of the statistical parameters including central tendency (e.g. means) or other basic estimates (e.g. regression coefficient) AND variation (e.g. standard deviation) or associated estimates of uncertainty (e.g. confidence intervals)
☐	☒	For null hypothesis testing, the test statistic (e.g. *F*, *t*, *r*) with confidence intervals, effect sizes, degrees of freedom and *P* value noted<br>*Give P values as exact values whenever suitable.*
☒	☐	For Bayesian analysis, information on the choice of priors and Markov chain Monte Carlo settings
☒	☐	For hierarchical and complex designs, identification of the appropriate level for tests and full reporting of outcomes
☒	☐	Estimates of effect sizes (e.g. Cohen's *d*, Pearson's *r*), indicating how they were calculated

Our web collection on statistics for biologists contains articles on many of the points above.

Software and code

Policy information about availability of computer code

Data collection	X-ray crystallographic data were collected on beamline BL19U1 at the Shanghai Synchrotron Radiation Facility (SSRF).Tyrosine phosphorylation assay was visualized by chemiluminescence imager ChemiDoc Touch (Bio-Rad) and the quantifications were performed using ImageJ program. Cell aggregation assay was observed with inverted fluorescence microscope Ti-S (Nikon).
Data analysis	Collected X-ray data were processed by HKL3000. Structure refinement and model building were performed with PHENIX and Coot. All models were validated with MolProbity. All structure figures were prepared with ChimeraX and PyMOL. Statistical analysis was performed using the GraphPad Prism 8 Software.

For manuscripts utilizing custom algorithms or software that are central to the research but not yet described in published literature, software must be made available to editors and reviewers. We strongly encourage code deposition in a community repository (e.g. GitHub). See the Nature Portfolio guidelines for submitting code & software for further information.

Data

Policy information about availability of data

All manuscripts must include a data availability statement. This statement should provide the following information, where applicable:

- Accession codes, unique identifiers, or web links for publicly available datasets
- A description of any restrictions on data availability
- For clinical datasets or third party data, please ensure that the statement adheres to our policy

Atomic coordinates and structure factors in this study have been deposited in the Protein Data Bank (PDB) under accession codes: 7Y54 [https://www.rcsb.org/structure/unreleased/7Y54] (α1-Ig1), 7Y4X [https://www.rcsb.org/structure/unreleased/7Y4X] (α7-Ig1), 7Y9A [https://www.rcsb.org/structure/unreleased/7Y9A] (β2v6-Ig1-2), 7Y95 [https://www.rcsb.org/structure/unreleased/7Y95] (β6v2-Ig1), 7Y6O [https://www.rcsb.org/structure/unreleased/7Y6O] (α25-Ig1-3), 7Y5J [https://www.rcsb.org/structure/unreleased/7Y5J] (α1v7-Ig1), 7Y73 [https://www.rcsb.org/structure/unreleased/7Y73] (β3v7-Ig1), 7Y8H [https://www.rcsb.org/structure/unreleased/7Y8H] (α7-FNIII1), 7Y5R [https://www.rcsb.org/structure/unreleased/7Y5R] (α7-FNIII2), 7Y8I [https://www.rcsb.org/structure/unreleased/7Y8I] (α7-FNIII3), 7Y6E [https://www.rcsb.org/structure/unreleased/7Y6E] (β2v6-FNIII2-3), and 7Y8S [https://www.rcsb.org/structure/unreleased/7Y8S] (β2v6-FNIII1-3). Source data are provided with this paper.

Human research participants

Policy information about studies involving human research participants and Sex and Gender in Research.

Reporting on sex and gender	n/a
Population characteristics	n/a
Recruitment	n/a
Ethics oversight	n/a

Note that full information on the approval of the study protocol must also be provided in the manuscript.

Field-specific reporting

Please select the one below that is the best fit for your research. If you are not sure, read the appropriate sections before making your selection.

☒ Life sciences ☐ Behavioural & social sciences ☐ Ecological, evolutionary & environmental sciences

For a reference copy of the document with all sections, see nature.com/documents/nr-reporting-summary-flat.pdf

Life sciences study design

All studies must disclose on these points even when the disclosure is negative.

Sample size	For statistical analysis by two-tailed Student’s t-test, at least 3 biological replicates were used.
Data exclusions	No data were excluded.
Replication	All attempts at replication were successful. The experiments, unless specified otherwise, were performed in triplicate.
Randomization	Randomization is not applicable as experiments did not involved any organisms or sample groups.
Blinding	No author was aware of the outcomes of the experiments.

Reporting for specific materials, systems and methods

We require information from authors about some types of materials, experimental systems and methods used in many studies. Here, indicate whether each material, system or method listed is relevant to your study. If you are not sure if a list item applies to your research, read the appropriate section before selecting a response.

Materials & experimental systems

n/a	Involved in the study
☐	☒ Antibodies
☐	☒ Eukaryotic cell lines
☒	☐ Palaeontology and archaeology
☒	☐ Animals and other organisms
☒	☐ Clinical data
☒	☐ Dual use research of concern

Methods

n/a	Involved in the study
☒	☐ ChIP-seq
☒	☐ Flow cytometry
☒	☐ MRI-based neuroimaging

Antibodies

Antibodies used

We used anti-Flag antibody (Smart Lifesciences, Cat. No. SLAB01) and anti-phosphotyrosine antibody (HuaBio, Cat. No. ET1704-20, Clone No. JA10-49).

Validation

For the anti-Flag antibody, the company website states that the mouse IgG2B recombined monoclonal antibodies specifically recognize the DYKDDDDK tag. Anti-Flag antibody was used at a 1:5,000 dilution for western blotting. For the anti-phosphotyrosine antibody, the company website states that the phospho-tyrosine monoclonal antibodies developed by Cell Signaling Technology are exceptionally sensitive tools for studying tyrosine phosphorylation and monitoring tyrosine kinase activity. Anti-phosphotyrosine antibody was used at a 1:100 dilution for western blotting. Both antibodies are applicable for Western Blot based on the company statement, and our results in this manuscript (Fig. 5b,c) showed that both antibodies are effective.

Eukaryotic cell lines

Policy information about cell lines and Sex and Gender in Research

Cell line source(s)

SF9 and High5 insect cell lines were bought from BLUEFBIO (Cat. No. BFN607200875 and BFN607200458)

Authentication

SF9 and High5 insect cell lines were authenticated by cell-size, adherent capability, and growth rate.

Mycoplasma contamination

All cell lines tested negative for mycoplasma contamination by GMyc-PCR Mycoplasma Test Kit (YEASEN, Cat. No. 40601ES10).

Commonly misidentified lines
(See ICLAC register)

No misidentified cell lines were used in this study.
